# Association between SPISE and NAFLD in patients with type 2 diabetes

**DOI:** 10.3389/fmed.2025.1454938

**Published:** 2025-01-22

**Authors:** Hongyan Zhao, Baolan Ji, Xin Wang, Shuwei Shi, Jie Sheng, Xuan Ma, Bo Ban, Guanqi Gao

**Affiliations:** ^1^School of Clinical Medicine, Shandong Second Medical University, Weifang, Shandong, China; ^2^Department of Endocrinology, Linyi People’s Hospital Affiliated to Shandong Second Medical University, Linyi, Shandong, China; ^3^Department of Endocrinology, Affiliated Hospital of Jining Medical University, Jining, Shandong, China

**Keywords:** type 2 diabetes, SPISE, NAFLD, insulin sensitivity, insulin resistance

## Abstract

**Aims:**

Non-alcoholic fatty liver disease (NAFLD) is closely related to type 2 diabetes (T2D), with reduced insulin sensitivity being a key factor in their disrupted metabolic processes. The single point insulin sensitivity estimator (SPISE) is a novel index. This study aims to explore the association between SPISE and NAFLD in T2D population.

**Methods:**

This study included a total of 2,459 patients with T2D. SPISE was calculated based on high density lipoprotein-cholesterol (HDL-c), triglycerides (TG), and body mass index (BMI). Participants were categorized into NAFLD and non-NAFLD groups based on the results of ultrasonographic diagnosis. The relationship between SPISE and NAFLD was analyzed separately for each gender.

**Results:**

The overall prevalence of NAFLD is 38.5%. In females and males, the SPISE was significantly reduced in the NAFLD group compared to the non-NAFLD group (both *P* < 0.05). The prevalence of NAFLD showed a significant reduction across quartiles of the SPISE in both genders (both *P* < 0.05).

Additionally, univariate correlation analysis showed a negative correlation between SPISE and NAFLD (both *P* < 0.05). In multivariate regression analysis, a reduced SPISE was identified as an independent risk factor for NAFLD (odds ratios of 0.572 and 0.737, 95% CI of 0.477–0.687 and 0.587–0.926, respectively).

Moreover, the area under the receiver operating characteristic (ROC) curve for SPISE was 0.209 in females and 0.268 in males (95% CI of 0.175–0.244 and 0.216–0.320, respectively). These results are more meaningful than those of other variables.

**Conclusion:**

SPISE is significantly reduced in NAFLD patients with T2D. Compared to other indicators, SPISE demonstrates superior predictive value in diagnosing NAFLD, and it is independent of gender.

## 1 Introduction

Non-alcoholic fatty liver disease (NAFLD) is a widespread health issue, with a worldwide prevalence of 25% (1). It has become the primary cause of chronic liver disease under the influence of type 2 diabetes (T2D) and obesity (2). Reports indicate that NAFLD has become the fastest-growing cause of liver-related deaths globally (3). Moreover, it is closely associated with the progression of chronic kidney disease (CKD) and cardiovascular disease (CVD) (4, 5). Many metabolic disorders not only affect the incidence of NAFLD but also increase the risk of its progression to non-alcoholic steatohepatitis (NASH), cirrhosis, hepatocellular carcinoma, and even death (6). And the link between T2D and NAFLD is thoroughly documented (7). Research indicates that T2D is associated with more than double the risk of advanced hepatopathy (8). Furthermore, a meta-analysis reported that the prevalence of NASH with T2D patients was approximately 37.3%, significantly higher than the prevalence of progressive NAFLD in the general population (9). Therefore, in clinical practice, it would be valuable to have a simple and inexpensive index that could screen for NAFLD among T2D patients.

Numerous studies indicate that reduced insulin sensitivity (Si) or insulin resistance (IR) is one of the key pathophysiological factors in NAFLD (10–12). The gold standard for measuring insulin sensitivity is the hyperinsulinemic-euglycemic clamp (13); however, due to its cost, time consumption, and invasiveness, it is not widely used in clinical practice. The single point insulin sensitivity estimator (SPISE) is an alternative index of IR calculated from high density lipoprotein-cholesterol (HDL-c), triglycerides (TG), and body mass index (BMI) (14). Research indicates a strong correlation between SPISE and the hyperinsulinemic-euglycemic clamp (15). Additionally, the SPISE index is closely related to metabolic syndrome (MetS), cardiovascular metabolic risk in adolescents, and the cardiovascular prognosis of patients with T2D (16–18). It is also worth mentioning that SPISE is not only considered an effective indicator for predicting diabetes development in obese children (19), but SPISE-5.4 has also been proven to be a good predictor of diabetes development (20). Recent studies have reported a significant reduction in the SPISE among adolescents with obesity-related NAFLD (21). Additionally, research from Japan indicates that a reduction in SPISE is associated with an increased risk of NAFLD (22). Research also suggested an association between SPISE and pediatric NAFLD; however, after adjusting for confounding factors, this association is no longer significant (23). Currently, there is scarce research on the relationship between SPISE and NAFLD among T2D patients. This study aims to clarify the link between SPISE and NAFLD in T2D patients and assess SPISE’s predictive potential for NAFLD in this population.

## 2 Materials and methods

### 2.1 Study participants

During the period from February 2020 to March 2023, we collected clinical data from patients with T2D who were treated at the Department of Endocrinology of the Linyi People’s Hospital, Shandong Province, China. Exclusion criteria included: (1) patients under the age of 18; (2) patients with liver or kidney dysfunction; (3) evidence of autoimmune hepatitis, viral hepatitis, drug-induced fatty liver, or other chronic liver diseases; (4) habitual drinkers who consume alcohol more than 5 days per week, equivalent to an average daily intake of 38 grams for males and 26 grams for females (24); (5) patients with incomplete clinical data. Ultimately, 2,459 eligible patients were included in the study.

### 2.2 Anthropometric and Biochemical measurements

Participant demographic information and clinical baseline data were collected, such as age, gender, duration of diabetes, and smoking and drinking habits. Height, weight, systolic blood pressure (SBP), and diastolic blood pressure (DBP) were measured and recorded. Morning fasting venous blood samples were collected to determine levels of alanine aminotransferase (ALT), aspartate aminotransferase (AST), gamma-glutamyl transferase (GGT), total cholesterol (TC), triglycerides (TG), high-density lipoprotein cholesterol (HDL-c), low-density lipoprotein cholesterol (LDL-c), serum creatinine (Scr), uric acid (UA), fasting plasma glucose (FBG); glycated hemoglobin (HbA1c, high performance liquid chromatography) and hemoglobin (Hb), were measured using a biochemical analyzer (Cobas c 702, Roche, Germany). Urinary albumin to creatinine ratio (UACR) was tested by an autoanalyzer (Beckman Coulter AU5821). Fasting insulin (FINS, direct chemiluminescence method) was measured by the fully automated sample processing system (Aptio Automation, SIEMENS, USA).

Bioelectrical impedance analysis (Omron DUALSCAN HDS-2000, Kyoto, Japan) was employed to assess visceral fat area (VFA) and subcutaneous fat area (SFA).

### 2.3 Definition of NAFLD

Fatty liver diagnosis begins with ultrasound imaging and is supplemented by clinical evaluation, including medical history and physical examination, with specific attention to alcohol intake. Additional factors such as viral hepatitis and medication use are assessed. Laboratory tests, particularly liver function tests, help rule out other fatty liver conditions, culminating in a definitive diagnosis of NAFLD.

**Parameter calculations**

1. Body mass index (BMI) = weight (kg) / height (m)^2^;
2. TG/HDL-c = TG (mmol/l) / HDL-c (mmol/l);
3. SPISE index = (600 × HDL-c [mg/dL]^0.185^) / (TG [mg/dL]^0.2^ × BMI [kg/m^2^]^1.338^) (14);
4. HOMA-IR = FPG (mmol/L) × FINS (IU/mL)/22.5 (25).

### 2.4 Statistical analysis

Statistical analyses were performed using SPSS 22.0 (SPSS Inc, Chicago, IL, USA). Normally distributed variables were described using mean ± SD and analyzed with independent samples T-tests. Non-normally distributed variables were described using medians (interquartile ranges) and analyzed with Mann-Whitney U tests. Analysis of variance (ANOVA) and Student–Newman–Keuls tests were performed for multiple and pairwise comparisons of normally distributed data, and Kruskal-Wallis 1-way ANOVA test for abnormal distributions. Categorical variables were presented as percentage (%) and assessed using chi-square tests. Independent factors influencing NAFLD were identified using Spearman’s correlation and logistic regression analyses. Significance was set at *P* < 0.05 (two-tailed). The SPISE index’s ability to predict NAFLD was evaluated via the receiver operating characteristic (ROC) curve analysis.

## 3 Results

### 3.1 Clinical and biochemical characteristics

As shown in Table 1, this study included 2459 patients with T2D, with a mean age of 57.10 ± 13.4 years. The overall incidence of NAFLD was 38.5%, with rates of 36.1% in females and 41.8% in males. Compared to males, females had higher levels of age, SBP, TC, LDL-c, HDL-c and SPISE, but lower proportion of smokers, BMI, VFA, SFA, DBP, TG/HDL-c ratio, ALT, AST, GGT, UA, Scr, and Hb (all *P* < 0.05). There were no significant differences in diabetes duration, TG, HbA1c, FPG, UACR, HOMA-IR and FINS between the two groups (all *P* > 0.05).

**TABLE 1:** Clinical and biochemical characteristics stratified by gender.

Variables	All	Female	Male	*P*
Number	2459	1441	1018	
Age (years)	57.10 ± 13.4	58.3 ± 13.3	55.4 ± 13.3	<0.001
diabetes duration (years)	7.0 (2.0 ∼ 13.0)	7.0 (2.0 ∼ 13.0)	8.0 (2.0 ∼ 13.0)	0.548
Smoking (*n*, %)	385 (15.7%)	9 (0.6%)	376 (37.0%)	<0.001
BMI (kg/m^2^)	25.40 ± 3.89	25.21 ± 3.99	25.68 ± 3.71	0.003
VFA (cm^2^)	89.00 (64.00 ∼ 119.00)	79.00 (58.00 ∼ 104.00)	106.00 (80.00 ∼ 133.00)	<0.001
SFA (cm^2^)	180.00 (138 ∼ 229.00)	176.50 (131.25 ∼ 228.00)	186.00 (148.00 ∼ 230.00)	0.001
SBP (mmHg)	129.7 ± 19.2	130.3 ± 19.8	128.8 ± 18.2	0.043
DBP (mmHg)	80.3 ± 11.9	79.0 ± 11.8	82.2 ± 11.8	<0.001
TC (mmol/L)	4.85 ± 1.33	4.99 ± 1.30	4.65 ± 1.33	<0.001
LDL-c (mmol/L)	3.08 ± 1.50	3.18 ± 1.72	2.94 ± 1.12	<0.001
TG (mmol/L)	1.41 (0.99 ∼ 2.09)	1.41 (0.99 ∼ 2.03)	1.41 (0.99 ∼ 2.20)	0.212
HDL-c (mmol/L)	1.18 ± 0.35	1.25 ± 0.37	1.08 ± 0.30	<0.001
TG / HDL-c ratio	1.25 (0.78 ∼ 2.05)	1.17 (0.74 ∼ 1.90)	1.39 (0.83 ∼ 2.34)	<0.001
HbA1c (%)	9.43 ± 2.28	9.40 ± 2.25	9.48 ± 2.32	0.383
FPG (mmol/L)	9.24 ± 4.03	9.24 ± 4.12	9.24 ± 3.91	0.969
ALT (U/L)	17.40 (12.80 ∼ 26.40)	16.15 (11.90 ∼ 24.50)	19.40 (14.10 ∼ 31.10)	<0.001
AST (U/L)	17.30 (14.00 ∼ 22.70)	16.90 (13.60 ∼ 22.30)	18.00 (14.60 ∼ 23.40)	<0.001
GGT (U/L)	21.00 (15.00 ∼ 32.00)	19.00 (14.00 ∼ 27.00)	26.00 (18.00 ∼ 41.00)	<0.001
UA (μmolL)	290.87 ± 101.22	269.75 ± 97.45	320.92 ± 98.91	<0.001
Scr (μmol/L)	66.80 ± 28.45	60.15 ± 27.89	76.32 ± 26.49	<0.001
UACR (mg/g)	12.10 (6.20 ∼ 47.50)	12.20 (6.60 ∼ 42.80)	11.70 (5.60 ∼ 54.00)	0.167
Hb (g/L)	138.82 ± 18.96	131.85 ± 16.17	148.66 ± 18.24	<0.001
FINS (μIU/mL)	16.70 (10.40 ∼ 22.94)	17.17 (10.71 ∼ 23.44)	15.77 (10.21 ∼ 21.55)	0.054
SPISE	6.10 (5.04 ∼ 7.39)	6.25 (5.22 ∼ 7.57)	5.87 (4.81 ∼ 7.08)	<0.001
HOMA-IR	6.40 (3.46 ∼ 9.78)	6.40 (3.52 ∼ 9.71)	6.37 (3.40 ∼ 10.19)	0.905
NAFLD (*n*, %)	946 (38.5%)	520 (36.1%)	426 (41.8%)	0.004

BMI, body mass index; VFA, visceral fat area; SFA, subcutaneous fat area; SBP, systolic blood pressure; DBP, diastolic blood pressure; TC, total cholesterol; LDL-c, low-density lipoprotein cholesterol; TG, triglyceride; HDL-c, high-density lipoprotein cholesterol; HbA1c, glycosylated hemoglobin; FPG, fasting plasma glucose; ALT, alanine aminotransferase; AST, aspartate aminotransferase; GGT, γ - glutamyl transpeptidase; UA, uric acid; Scr, serum creatinine; UACR, urinary albumin to creatinine ratio; Hb, hemoglobin; FINS, fasting insulin; SPISE, the single point insulin sensitivity estimator; HOMA-IR, homeostatic model assessment for insulin resistance; NAFLD, non-alcoholic fatty liver disease. Data were presented as mean ± SD for normally distributed variables, and median (interquartile ranges) for abnormal distributions. Independent-Samples T test and Mann-Whitney U test were used for comparisons of normally and abnormally distributed continuous variables between male and female groups, respectively. Categorical variables were presented as percentage (%), and were compared by chi-square test. Statistical differences were defined by *P* (two-tailed) less than 0.05.

As shown in Table 2, for each gender, subjects were divided into two groups, including non-NAFLD and NAFLD groups, and the levels of each variable were compared. For females, compared to the non-NAFLD group, the NAFLD group showed significantly increased BMI, VFA, SFA, SBP, DBP, TC, LDL-c, TG, TG/HDL-c ratio, FPG, ALT, AST, GGT, UA, Hb, FINS and HOMA-IR (all *P* < 0.05), while age, diabetes duration, HDL-c, Scr, UACR and SPISE were significantly decreased (all *P* < 0.05). For males, the proportion of smokers and the levels of BMI, VFA, SFA, SBP, DBP, TC, LDL-c, TG, TG/HDL-c ratio, FBG, ALT, AST, GGT, UA, Hb and HOMA-IR were higher in the NAFLD group compared to the non-NAFLD group (all *P* < 0.05), while age, diabetes duration, HDL-c, Scr, UACR and SPISE were lower (all *P* < 0.05).

**TABLE 2:** Comparison of clinical and biochemical characteristics between non-NAFLD and NAFLD groups.

Variables	Female			Male		
	**Non-NAFLD group**	**NAFLD group**	***P***	**Non-NAFLD group**	**NAFLD group**	***P***
Number	921	520		592	426	
Age (years)	59.38 ± 12.91	56.38 ± 13.83	<0.001	58.33 ± 12.90	51.37 ± 12.89	<0.001
Diabetes duration (years)	8.0 (3.0 ∼ 15.0)	5.0 (1.0 ∼ 10.0)	<0.001	10.0 (4.0 ∼ 15.0)	5.0 (2.0 ∼ 10.0)	<0.001
Smoking (%)	5 (0.5%)	8 (0.8%)	0.730	203 (34.3%)	173 (40.6%)	0.048
BMI (kg/m^2^)	24.07 ± 3.64	27.23 ± 3.80	<0.001	24.45 ± 3.42	27.37 ± 3.43	<0.001
VFA (cm^2^)	67.00 (48.00 ∼ 90.00)	97.00 (77.00 ∼ 120.50)	<0.001	92.00 (64.75 ∼ 121.00)	124.00 (101.00 ∼ 151.00)	<0.001
SFA (cm^2^)	155.00(116.00 ∼ 200.00)	214.00 (171.00 ∼ 261.00)	<0.001	168.00 (129.00 ∼ 202.00)	211.00 (175.00 ∼ 255.50)	<0.001
SBP (mmHg)	128.84 ± 20.30	132.97 ± 18.59	<0.001	127.79 ± 19.45	130.10 ± 16.26	0.040
DBP (mmHg)	77.32 ± 11.65	82.02 ± 11.52	<0.001	80.06 ± 11.66	85.11 ± 11.35	<0.001
TC (mmol/L)	4.87 ± 1.32	5.20 ± 1.26	<0.001	4.48 ± 1.26	4.89 ± 1.39	<0.001
LDL-c (mmol/L)	3.06 ± 1.35	3.39 ± 2.21	<0.001	2.89 ± 1.14	3.05 ± 1.08	0.006
TG (mmol/L)	1.25 (0.88 ∼ 1.74)	1.72 (1.28 ∼ 2.55)	<0.001	1.21 (0.85 ∼ 1.79)	1.79 (1.26 ∼ 2.80)	<0.001
HDL-c (mmol/L)	1.29 ± 0.38	1.17 ± 0.33	<0.001	1.12 ± 0.28	1.03 ± 0.31	<0.001
TG / HDL-c ratio	1.01 (0.64 ∼ 1.59)	1.55 (1.04 ∼ 2.37)	<0.001	1.11 (0.70 ∼ 1.80)	1.83 (1.20 ∼ 3.07)	<0.001
HbA1c (%)	9.33 ± 2.36	9.52 ± 2.03	0.118	9.52 ± 2.48	9.42 ± 2.08	0.531
FPG (mmol/L)	8.91 ± 4.22	9.83 ± 3.87	<0.001	9.02 ± 4.28	9.56 ± 3.30	0.026
ALT (U/L)	14.60 (10.90 ∼ 22.00)	19.15 (14.20 ∼ 29.75)	<0.001	17.40 (13.20 ∼ 25.10)	23.70 (16.20 ∼ 38.30)	<0.001
AST (U/L)	16.40 (13.18 ∼ 21.33)	17.70 (14.23 ∼ 24.68)	<0.001	17.30 (14.00 ∼ 21.43)	19.00 (15.20 ∼ 26.45)	<0.001
GGT (U/L)	16.95 (12.00 ∼ 23.00)	24.00 (17.00 ∼ 33.00)	<0.001	21.00 (16.00 ∼ 31.00)	33.00 (24.00 ∼ 53.00)	<0.001
UA (μmolL)	257.23 ± 94.17	291.77 ± 99.30	<0.001	310.61 ± 102.19	335.26 ± 92.40	<0.001
Scr (μmol/L)	62.20 ± 32.20	56.54 ± 17.39	<0.001	78.64 ± 30.54	73.12 ± 19.18	<0.001
UACR (mg/g)	12.85 (6.70 ∼ 58.13)	11.40 (6.40 ∼ 30.20)	0.010	13.90 (5.80 ∼ 83.40)	9.30 (5.10 ∼ 36.60)	0.002
Hb (g/L)	129.29 ± 16.77	136.36 ± 13.98	<0.001	144.36 ± 20.26	154.71 ± 12.72	<0.001
FINS (μIU/mL)	16.57 (8.44 ∼ 23.18)	18.41 (13.27 ∼ 24.47)	0.002	15.00 (9.14 ∼ 22.33)	16.44 (11.29 ∼ 21.27)	0.263
SPISE	6.87 (5.79 ∼ 8.20)	5.35 (4.57 ∼ 6.21)	<0.001	6.48 (5.50 ∼ 7.91)	5.12 (4.33 ∼ 5.95)	<0.001
HOMA-IR	5.71 (2.91 ∼ 9.12)	7.57 (4.81 ∼ 10.53)	<0.001	5.53 (2.90 ∼ 10.21)	6.92 (3.94 ∼ 10.13)	0.009

BMI, body mass index; VFA, visceral fat area; SFA, subcutaneous fat area; SBP, systolic blood pressure; DBP, diastolic blood pressure; TC, total cholesterol; LDL-c, low-density lipoprotein cholesterol; TG, triglyceride; HDL-c, high-density lipoprotein cholesterol; HbA1c, glycosylated hemoglobin; FPG, fasting plasma glucose; ALT, alanine aminotransferase; AST, aspartate aminotransferase; GGT, γ - glutamyl transpeptidase; UA, uric acid; Scr, serum creatinine; UACR, urinary albumin to creatinine ratio; Hb, hemoglobin; FINS, fasting insulin; SPISE, the single point insulin sensitivity estimator; HOMA-IR, homeostatic model assessment for insulin resistance; NAFLD, non-alcoholic fatty liver disease; Data were presented as mean ± SD for normally distributed variables, and median (interquartile ranges) for abnormal distributions. Independent-Samples T test and Mann-Whitney U test were used for comparisons of normally and abnormally distributed continuous variables between non-NAFLD and NAFLD groups, respectively. Categorical variables were presented as percentage (%), and were compared by chi-square test. Statistical differences were defined by *P* (two-tailed) less than 0.05.

As shown in Table 3, male and female patients were separately divided into four groups according to the quartiles of the SPISE: Q1 group (female: 2.58–5.22; male: 2.25–4.81), Q2 group (female: 5.22–6.25; male: 4.81–5.87), Q3 group (female: 6.25–7.57; male: 5.87–7.08), and Q4 group (female: 7.57–14.52; male: 7.08–15.05). For the females, as the quartiles of SPISE increased, the duration of diabetes, HDL-c showed a gradual increased, while the age, BMI, VFA, SFA, SBP, DBP, TC, LDL-c, TG, TG/HDL-c, FPG, ALT, AST, GGT, UA, Hb, FINS, HOMA-IR and the incidence of NAFLD exhibited a gradual decreased (all *P* < 0.05). There was no statistically significant difference in the proportion of smokers, HbA1c, Scr and UACR among the four groups (all *P* > 0.05). For the males, as the quartiles of SPISE increased, the age, duration of diabetes, HDL-c showed a gradual increased, while the proportion of smokers, BMI, VFA, SFA, SBP, DBP, TC, LDL-c, TG, TG/HDL-c, HbA1c, FPG, ALT, AST, GGT, UA, UACR, Hb, HOMA-IR and the incidence of NAFLD exhibited a gradual decreased (all *P* < 0.05). There was no statistically significant difference in Scr and FINS among the four groups (all *P* > 0.05).

**TABLE 3:** Comparison of variables according to the categories of the SPISE.

Variables	Female					Male				
	**Q1**	**Q2**	**Q3**	**Q4**	**P**	**Q1**	**Q2**	**Q3**	**Q4**	**P**
Age (years)	56.32 ± 15.30	59.80 ± 12.30^a^	59.37 ± 11.92^a^	57.72 ± 13.25^b^	0.001	48.30 ± 13.22	55.44 ± 12.82^a^	59.14 ± 11.24^ab^	58.81 ± 13.14^ab^	<0.001
Diabetes duration (years)	6.0 (2.0∼10.0)	8.0(3.0∼13.0)^a^	8.0(3.0∼15.0)^a^	8.0(2.0∼13.0)	0.020	5.0(2.0∼10.0)	8.0(3.0∼13.0)^a^	10.0(3.0∼15.0)^a^	8.0(3.0∼15.0)^ab^	<0.001
Smoking (n, %)	1(0.3%)	4(1.1%)	0(0%)	4(1.1%)	0.123	110(43.5%)	100(38.9%)	91(35.8%)	75(29.6%)	0.012
BMI (kg/m^2^)	29.81 ± 3.63	25.94 ± 1.85^a^	23.95 ± 1.79^ab^	21.09 ± 1.90^abc^	<0.001	29.66 ± 3.19	26.65 ± 1.71^a^	24.74 ± 1.69^ab^	21.62 ± 2.28^abc^	<0.001
VFA (cm^2^)	114.50 (91.00∼138.00)	88.00^a^ (74.00∼104.00)	71.00^ab^ (59.00∼90.00)	46.50^abc^ (30.00∼64.00)	<0.001	140.00 (118.75∼166.25)	119.00^a^ (97.00∼139.00)	97.50^ab^ (79.00∼117.25)	63.50^abc^ (38.00∼83.00)	<0.001
SFA (cm^2^)	246.50 (206.75∼294.25)	195.00^a^ (160.50∼228.50)	162.00^ab^ (134.00∼188.00)	113.50^abc^ (80.00∼144.00)	<0.001	242.00 (206.00∼287.75)	196.00^a^ (170.00∼232.00)	179.00^ab^ (148.00∼200.00)	124.50^abc^ (99.75∼162.00)	<0.001
SBP (mmHg)	133.96 ± 19.42	132.17 ± 18.62	129.97 ± 19.70^a^	125.22 ± 20.38^abc^	<0.001	133.31 ± 18.03	128.48 ± 17.30^a^	129.05 ± 18.49^a^	124.18 ± 17.95^abc^	<0.001
DBP (mmHg)	82.36 ± 12.00	79.82 ± 11.27^a^	78.18 ± 11.89^a^	75.68 ± 11.10^abc^	<0.001	86.85 ± 12.01	81.80 ± 10.51^a^	81.90 ± 11.72 ^a^	78.15 ± 11.30 ^abc^	<0.001
TC (mmol/L)	5.20 ± 1.36	4.98 ± 1.28 ^a^	4.85 ± 1.30 ^a^	4.91 ± 1.25 ^a^	0.002	5.02 ± 1.53	4.69 ± 1.29 ^a^	4.47 ± 1.16 ^ab^	4.42 ± 1.25 ^ab^	<0.001
LDL-c (mmol/L)	3.26 ± 1.67	3.35 ± 2.58	3.05 ± 1.05 ^b^	3.04 ± 1.10 ^b^	0.035	2.93 ± 1.10	3.11 ± 1.32 ^a^	2.95 ± 0.99 ^b^	2.77 ± 1.02 ^b^	0.009
TG (mmol/L)	2.23 (1.58∼3.14)	1.62 ^a^ (1.25∼2.09)	1.26 ^ab^ (0.98∼1.60)	0.87 ^abc^ (0.68∼1.14)	<0.001	2.90 (2.02∼4.56)	1.65 ^a^ (1.28∼2.14)	1.22 ^ab^ (0.99∼1.54)	0.83 ^abc^ (0.66∼1.07)	<0.001
HDL-c (mmol/L)	1.05 ± 0.25	1.16 ± 0.25 ^a^	1.30 ± 0.36 ^ab^	1.49 ± 0.43 ^abc^	<0.001	0.89 ± 0.23	1.01 ± 0.18 ^a^	1.11 ± 0.22 ^ab^	1.32 ± 0.35 ^abc^	<0.001
TG / HDL-c ratio	2.19 (1.46∼3.22)	1.42 ^a^ (1.06∼1.92)	1.01 ^ab^ (0.74∼1.42)	0.62 ^abc^ (0.43∼0.87)	<0.001	3.24 (2.18∼5.59)	1.68 ^a^ (1.25∼2.23)	1.15 ^ab^ (0.85∼1.48)	0.66 ^abc^ (0.50∼0.90)	<0.001
HbA1c, n (%)	9.43 ± 1.98	9.24 ± 2.06 ^a^	9.42 ± 2.46 ^a^	9.48 ± 2.45 ^bc^	0.532	9.70 ± 2.24	9.20 ± 2.10	9.16 ± 2.13	9.86 ± 2.71	0.001
FPG (mmol/L)	10.00 ± 3.78	9.11 ± 4.09 ^a^	9.00 ± 4.24 ^a^	8.83 ± 4.27 ^a^	0.001	10.12 ± 3.36	9.31 ± 3.59	8.61 ± 3.46 ^ab^	8.94 ± 4.88 ^a^	<0.001
ALT (U/L)	19.10 (13.33∼30.90)	15.80 ^a^ (12.15∼24.35)	15.20 ^a^ (11.20∼21.93)	15.20 ^a^ (11.00∼23.00)	<0.001	24.10 (16.70∼40.05)	20.00 ^a^ (14.90∼31.00)	18.35 ^a^ (13.70∼26.55)	17.00 ^a^ (12.60∼24.50)	<0.001
AST (U/L)	17.40 (14.30∼25.65)	16.60 ^a^ (13.53∼21.20)	16.20 ^a^ (13.10∼21.10)	17.10 ^a^ (13.70∼22.33)	0.003	19.30 (15.50∼28.70)	17.50 ^a^ (14.10∼22.95)	17.40 ^a^ (14.13∼21.78)	17.80 ^a^ (14.10∼22.58)	<0.001
GGT (U/L)	25.00 (17.00∼36.05)	19.00 ^a^ (14.00∼27.00)	17.80 ^a^ (13.00∼24.00)	15.00 ^abc^ (12.00∼21.00)	<0.001	35.80 (26.00∼56.75)	27.00 ^a^ (20.55∼41.00)	24.00 ^a^ (18.00∼35.00)	19.00 ^abc^ (14.00∼28.00)	<0.001
UA (μmol/L)	309.19 ± 99.23	270.56 ± 92.10 ^a^	252.96 ± 86.42 ^ab^	245.67 ± 99.05 ^ab^	<0.001	364.28 ± 100.97	325.67 ± 90.46 ^a^	304.37 ± 87.60 ^ab^	289.40 ± 100.36 ^ab^	<0.001
Scr (μmol/L)	60.23 ± 21.89	60.26 ± 31.84	61.38 ± 32.56	58.75 ± 23.83	0.659	76.27 ± 27.85	76.84 ± 28.34	77.83 ± 26.61	74.29 ± 22.79	0.500
UACR (mg/g)	13.60 (6.90∼42.90)	11.30 (6.20∼33.45)	11.70 (6.88∼42.33)	12.05 (6.45∼53.65)	0.318	15.10 (6.40∼94.70)	9.15 (4.93∼33.23)	8.60 (4.65∼44.35)	14.00 (6.20∼73.10)	0.001
Hb (g/L)	134.96 ± 14.93	133.09 ± 15.95	130.54 ± 16.79 ^ab^	128.85 ± 16.34^ab^	<0.001	153.85 ± 15.99	151.78 ± 16.87	148.85 ± 17.59 ^a^	140.21 ± 19.40 ^abc^	<0.001
FINS (μIU/mL)	19.34 (14.75∼25.47)	17.23 (10.96∼23.51)	16.84 ^a^ (9.90∼23.18)	15.02 ^a^ (6.12∼21.76)	<0.001	17.81 (12.02∼22.09)	15.49 (10.64∼19.98)	14.89 (9.10∼20.64)	14.85 (9.33∼21.61)	0.071
HOMA-IR	8.48 (5.30∼11.94)	6.58 ^a^ (3.58∼9.46)	5.76 ^a^ (3.50∼9.39)	5.03 ^ab^ (2.02∼8.22)	<0.001	7.70 (4.33∼10.69)	6.96 ^a^ (3.62∼10.22)	5.24 ^a^ (3.24∼8.43)	5.14 ^a^ (2.75∼8.61)	0.001
NAFLD, n (%)	241(66.2%)	156(43.6%)	88(24.4%)	35(9.7%)	<0.001	176(69.3%)	138(53.7%)	81(31.9%)	31(12.3%)	<0.001

BMI, body mass index; VFA, visceral fat area; SFA, subcutaneous fat area; SBP, systolic blood pressure; DBP, diastolic blood pressure; TC, total cholesterol; LDL-c, low-density lipoprotein cholesterol; TG, triglyceride; HDL-c, high-density lipoprotein cholesterol; HbA1c, glycosylated hemoglobin; FPG, fasting plasma glucose; ALT, alanine aminotransferase; AST, aspartate aminotransferase; GGT, γ - glutamyl transpeptidase; UA, uric acid; Scr, serum creatinine; UACR, urinary albumin to creatinine ratio; Hb, hemoglobin; FINS, fasting insulin; SPISE, the single point insulin sensitivity estimator; HOMA-IR, homeostatic model assessment for insulin resistance; NAFLD, non-alcoholic fatty liver disease. Data were presented as mean ± SD for normally distributed variables, and median (interquartile ranges) for abnormal distributions. Analysis of variance (ANOVA) and Student–Newman–Keuls tests were performed for multiple and pairwise comparisons of normally distributed data, and Kruskal-Wallis 1-way ANOVA test for abnormal distributions. Categorical variables were presented as percentage (%) and were compared by chi-square test. Statistical differences were defined by P (two-tailed) less than 0.05.

*^a^P* < 0.05 versus Q1;^ b^*P* < 0.05 Q3 versus Q2;^ c^*P* < 0.05 Q4 versus Q3.

### 3.2 Univariate analysis

As shown in Table 4, the relationship between NAFLD and each variable was analyzed using Spearman’s correlation analysis. In females, the results indicated that NAFLD was positively correlated with BMI, VFA, SFA, SBP, DBP, TC, LDL-c, TG, TG/HDL-c, HbA1c, FBG, ALT, AST, GGT, UA, Hb, FINS and HOMA-IR (all *P* < 0.05), and negatively correlated with age, duration of diabetes, HDL-c, Scr, UACR, and SPISE (all *P* < 0.05). In males, the proportion of smokers, BMI, VFA, SFA, SBP, DBP, TC, LDL-c, TG, TG/HDL-c, FBG, ALT, AST, GGT, UA, Hb and HOMA-IR were positively correlated with NAFLD, while age, duration of diabetes, HDL-c, UACR and SPISE were negatively correlated (all *P* < 0.05). In females, there was no significant relationship between NAFLD and the proportion of smokers (all *P* > 0.05), and in males, there was no apparent relationship between NAFLD and HbA1c, Scr and FINS (all *P* > 0.05).

**TABLE 4:** The correlation between NAFLD and different variables by univariate analysis.

Variables	Female		Male	
	**For NAFLD**		**For NAFLD**	
	**Correlation coefficient**	***p***	**Correlation coefficient**	***p***
Age	−0.096	<0.001	−0.267	<0.001
Diabetes duration	−0.177	<0.001	−0.244	<0.001
Smoking	0.014	0.602	0.064	0.041
BMI	0.396	<0.001	0.405	<0.001
VFA	0.414	<0.001	0.414	<0.001
SFA	0.403	<0.001	0.395	<0.001
SBP	0.110	<0.001	0.085	0.007
DBP	0.205	<0.001	0.223	<0.001
TC	0.137	<0.001	0.155	<0.001
LDL-c	0.135	<0.001	0.110	<0.001
TG	0.323	<0.001	0.336	<0.001
HDL-c	−0.191	<0.001	−0.198	<0.001
TG / HDL-c ratio	0.314	<0.001	0.331	<0.001
HbA1c	0.070	0.010	−0.004	0.891
FPG	0.148	<0.001	0.131	<0.001
ALT	0.248	<0.001	0.270	<0.001
AST	0.123	<0.001	0.150	<0.001
GGT	0.329	<0.001	0.379	<0.001
UA	0.194	<0.001	0.154	<0.001
Scr	−0.058	0.028	−0.059	0.061
UACR	−0.069	0.010	−0.099	0.002
Hb	0.214	<0.001	0.269	<0.001
FINS	0.108	0.002	0.048	0.263
SPISE	−0.450	<0.001	−0.441	<0.001
HOMA-IR	0.176	<0.001	0.113	0.009

BMI, body mass index; VFA, visceral fat area; SFA, subcutaneous fat area; SBP, systolic blood pressure; DBP, diastolic blood pressure; TC, total cholesterol; LDL-c, low-density lipoprotein cholesterol; TG, triglyceride; HDL-c, high-density lipoprotein cholesterol; HbA1c, glycosylated hemoglobin; FPG, fasting plasma glucose; ALT, alanine aminotransferase; AST, aspartate aminotransferase; GGT, γ - glutamyl transpeptidase; UA, uric acid; Scr, serum creatinine; UACR, urinary albumin to creatinine ratio; Hb, hemoglobin; FINS, fasting insulin; SPISE, the single point insulin sensitivity estimator; HOMA-IR, homeostatic model assessment for insulin resistance; NAFLD, non-alcoholic fatty liver disease; Correlation coefficients between NAFLD and different variables were determined by Spearman’s correlation analysis.

### 3.3 Logistic regression analysis

Using NAFLD as the dependent variable, based on the results of Spearman’s correlation analysis, the independent variables included age, diabetes duration, HDL-c, Scr, UACR, SPISE, BMI, VFA, SFA, SBP, DBP, TC, LDL-c, TG, TG/HDL-c, HbA1c, FBG, ALT, AST, GGT, UA, Hb, FINS and HOMA-IR for females, and the proportion of smokers, age, diabetes duration, HDL-c, UACR, SPISE, BMI, VFA, SFA, SBP, DBP, TC, LDL-c, TG, TG/HDL-c, FBG, ALT, AST, GGT, UA, Hb and HOMA-IR for males. A binary logistic regression analysis was conducted to identify the independent correlates of NAFLD (Table 5). The results indicated that in females, SPISE (OR: 0.572, 95% CI 0.477–0.687), VFA (OR: 1.009, 95% CI 1.001–1.017), FPG (OR: 1.059, 95% CI 1.002–1.120), DBP (OR: 1.026, 95% CI 1.006–1.046), UA (OR: 1.005, 95% CI 1.002–1.008), TC (OR: 1.236, 95% CI 1.036–1.475), and Scr (OR: 0.973, 95% CI 0.958–0.988) were independently associated with NAFLD, while in males, SPISE (OR: 0.737, 95% CI 0.587–0.926), VFA (OR: 1.013, 95% CI 1.005–1.021), diabetes duration (OR: 0.940, 95% CI 0.903–0.978), Hb (OR: 1.030, 95% CI 1.013–1.047), and GGT (OR: 1.009, 95% CI 1.002–1.016) were independently related to NAFLD.

**TABLE 5:** The relative risk for NAFLD by logistic regression analysis.

Variables	B	SE	Wald	*P*	OR	95.0 % CI for OR
**Female**						
SPISE	−0.558	0.093	36.165	<0.001	0.572	0.477–0.687
VFA	0.009	0.004	5.252	0.022	1.009	1.001–1.017
FPG	0.057	0.028	4.087	0.043	1.059	1.002–1.120
DBP	0.025	0.01	6.672	0.01	1.026	1.006–1.046
UA	0.005	0.001	13.628	<0.001	1.005	1.002–1.008
TC	0.212	0.09	5.535	0.019	1.236	1.036–1.475
Scr	−0.027	0.008	11.79	0.001	0.973	0.958–0.988
**Male**						
SPISE	−0.305	0.116	6.856	0.009	0.737	0.587–0.926
VFA	0.013	0.004	9.581	0.002	1.013	1.005–1.021
Diabetes duration	−0.062	0.02	9.214	0.002	0.94	0.903–0.978
Hb	0.03	0.008	12.353	<0.001	1.03	1.013–1.047
GGT	0.009	0.004	5.743	0.017	1.009	1.002–1.016

NAFLD, non-alcoholic fatty liver disease; SPISE, the single point insulin sensitivity estimator; VFA, visceral fat area; FPG, fasting plasma glucose; DBP, diastolic blood pressure; UA, uric acid; TC, total cholesterol; Scr, serum creatinine; Hb, hemoglobin; GGT,γ- glutamyl transpeptidase; SE, standard error; CI, confidence interval; OR, odd ratio.

### 3.4 Areas under the ROC curve analysis

Finally, based on the variables that entered the model last, the formula used to calculate SPISE and the insulin resistance-related indicators, the predictive capabilities of SPISE, HDL-c, diabetes duration, Scr, VFA, BMI, GGT, ALT, TG, TG/HDL-c ratio, HOMA-IR, Hb, UA, and TC for NAFLD were evaluated separately for different genders (Table 6). The results showed that in females, the area under the ROC curve for SPISE was 0.209 (95% CI 0.175–0.244, *P* < 0.001), and in males, it was 0.268 (95% CI 0.216–0.320, *P* < 0.001), both of which were superior to the other variables.

**TABLE 6:** Analysis of areas under the ROC curves for predicting NAFLD.

Female				Male		
Variables	Area	SE	95.0 % CI	Area	SE	95.0 % CI
SPISE	0.209	0.017	0.175–0.244	0.268	0.027	0.216–0.320
HDL-c	0.364	0.022	0.570–0.654	0.390	0.030	0.331–0.449
Diabetes duration	0.421	0.023	0.376–0.466	0.369	0.029	0.312–0.427
Scr	0.458	0.023	0.412–0.503	0.432	0.030	0.373–0.492
VFA	0.756	0.018	0.720–0.792	0.723	0.027	0.671–0.775
BMI	0.762	0.018	0.726–0.799	0.713	0.027	0.659–0.766
GGT	0.724	0.020	0.686–0.763	0.744	0.026	0.693–0.795
ALT	0.612	0.022	0.569–0.655	0.656	0.029	0.599–0.713
TG	0.697	0.021	0.657–0.738	0.681	0.028	0.626–0.736
TG / HDL-c ratio	0.694	0.021	0.653–0.734	0.675	0.028	0.619–0.731
HOMA-IR	0.612	0.022	0.570–0.654	0.555	0.031	0.495–0.615
Hb	0.618	0.022	0.575–0.662	0.684	0.028	0.628–0.739
UA	0.629	0.022	0.587–0.672	0.570	0.030	0.511–0.630
TC	0.599	0.022	0.555–0.644	0.608	0.030	0.549–0.666

NAFLD, non-alcoholic fatty liver disease; SPISE, the single point insulin sensitivity estimator; HDL-c, high-density lipoprotein cholesterol; Scr, serum creatinine; VFA, visceral fat area; BMI, body mass index; GGT, γ- glutamyl transpeptidase; ALT, alanine aminotransferase; TG, triglyceride; HOMA-IR, homeostatic model assessment for insulin resistance; Hb, hemoglobin; UA, uric acid; TC, total cholesterol; SE, standard error; CI, confidence interval.

## 4 Discussion

This study found that SPISE was independently associated with NAFLD in T2D population, with no gender differences observed. Additionally, SPISE demonstrated a clear advantage in predicting NAFLD within this population.

NAFLD as the most prevalent liver disease, exhibits an increasing trend in incidence (26). Reports indicated a strong correlation between T2D and NAFLD: the incidence of NAFLD and NASH was particularly pronounced in individuals diagnosed with T2D (9); the existence of NAFLD raised the risk of T2D development by five times (27). In this study, the overall incidence of NAFLD was 38.5%, which is higher than the global incidence rate, further validating the aforementioned perspective (1). Therefore, the high prevalence of NAFLD in T2D population warrants attention. Currently, the routine method for diagnosing NAFLD in clinical practice is through ultrasound. However, due to its time-consuming and labor-intensive nature, it is not suitable for large-scale epidemiological studies. SPISE is an insulin sensitivity index based on lipids and BMI, our study found that it is closely related to traditional IR indicators, including HOMA-IR and the TG/HDL-c ratio. As the SPISE quartiles increased, both HOMA-IR and the TG/HDL-c ratio were gradually decreased. Additionally, some studies had found that the SPISE demonstrated higher accuracy in predicting MetS and IR compared to other measures such as the TG/HDL-c ratio and HOMA-IR (14, 28). Extensive research had confirmed that NAFLD was closely associated with insulin resistance and metabolic syndrome (10, 11, 29, 30). Recent studies have reported that SPISE was closely associated with NAFLD related to adolescent obesity and NAFLD in healthy screening participants (21, 22). However, there is currently a lack of evidence for SPISE as a predictor of NAFLD in T2D population.

Our study corroborated the capability of SPISE to predict NAFLD among T2D population. HOMA-IR and the TG/HDL-c ratio were also closely related to NAFLD (31, 32), and therefore we included these IR-related indicators in our study. The results showed that they did not enter the regression model, and compared to SPISE, their area under the ROC curve was significantly smaller. A Japanese study similarly found that a 1.8-fold increased risk of concurrent NAFLD and T2D was associated with SPISE, aligning with our findings (22). However, that study included only 58 patients with both NAFLD and T2D, whereas our study involved 2,459 T2D patients with NAFLD. Additionally, we conducted gender-stratified analyses, which yielded consistent results, further substantiating the predictive power of SPISE in this group. Beyond IR, dyslipidemia and obesity are also significant factors related to NAFLD (33). SPISE, as a comprehensive indicator that includes metrics related to lipids and obesity, is convenient, accessible and low-cost, making it highly suitable for large-scale clinical application.

In addition, the results of this study indicated that NAFLD was closely associated with VFA in both males and females. This is generally consistent with previous research findings (34). GGT, ALT and AST are liver enzymes closely associated with NAFLD and NASH (35–37). In our analysis of female samples using Spearman’s correlation, AST, GGT, and ALT all showed positive correlations with NAFLD, yet these variables were not included in the final binary logistic regression model. In contrast, in male samples, GGT was incorporated into the regression model. However, the predictive power of the liver enzyme included in the final regression model, as indicated by the area under the ROC curve, remained inferior to that of the SPISE index. This gender discrepancy may stem from differences in research methodologies and sample selection criteria.

## 5 Limitations

This study faces several limitations. Firstly, due to its cross-sectional design, we cannot establish a causal relationship between the SPISE index and NAFLD. Secondly, the diagnosis of NAFLD was not made using the gold standard of liver biopsy, which may lead to diagnostic bias. Lastly, as this study was conducted at a single center, future research should be multi-center in order to further validate our findings and the replication of the study.

## 6 Conclusion

This study demonstrated that SPISE may have potential advantages over other commonly used biomarkers in identifying NAFLD among T2D patients. As a simple insulin sensitivity index, the specific utility of SPISE in predicting NAFLD among T2D patients remains to be further investigated.

## Data Availability

The original contributions presented in this study are included in this article/supplementary material, further inquiries can be directed to the corresponding authors.
